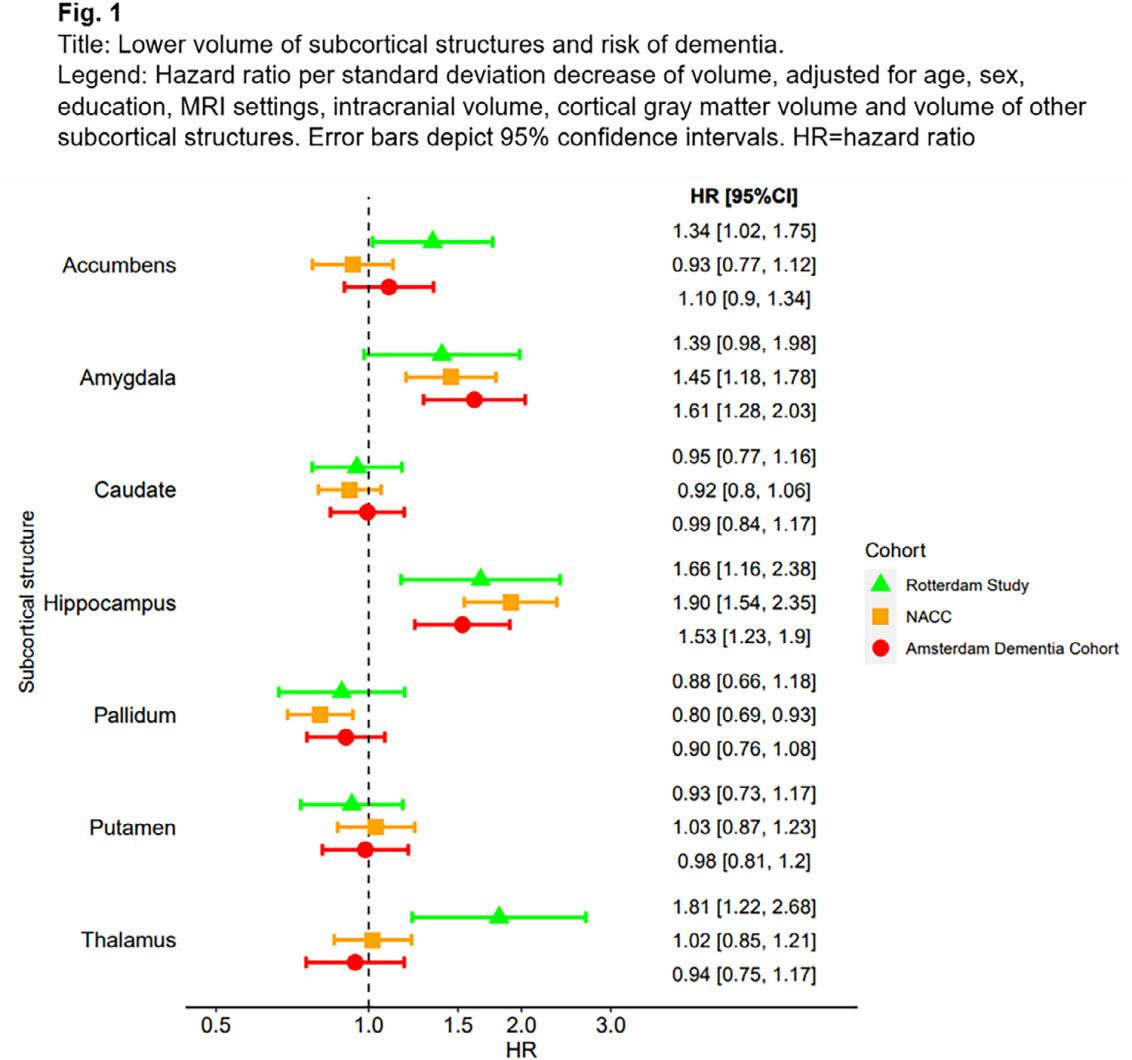# Unravelling the role of subcortical brain volumes in the conversion to dementia: a multi‐cohort analysis

**DOI:** 10.1002/alz.090756

**Published:** 2025-01-09

**Authors:** Mathijs T. Rosbergen, Pieter J. van der Veere, Jacqueline Josephine Claus, Tavia E Evans, Vikram Venkatraghavan, Frederik Barkhof, Argonde C. van Harten, M. Arfan Ikram, Wiesje M. van der Flier, Meike W. Vernooij, Frank J. Wolters

**Affiliations:** ^1^ Erasmus MC University Medical Center, Rotterdam, Zuid Holland Netherlands; ^2^ Amsterdam Neuroscience, Neurodegeneration, Amsterdam Netherlands; ^3^ Alzheimer Center Amsterdam, Neurology, Vrije Universiteit Amsterdam, Amsterdam UMC location VUmc, Amsterdam Netherlands; ^4^ Department of Epidemiology and Data Science, Amsterdam UMC, Amsterdam Netherlands; ^5^ Erasmus Medical Center, Rotterdam, Zuid‐Holland Netherlands; ^6^ Department of Radiology and Nuclear Medicine, Erasmus University Medical Center, Rotterdam Netherlands; ^7^ Erasmus University Medical Center, Rotterdam Netherlands; ^8^ Alzheimer Center Amsterdam, Department of Neurology, Amsterdam Neuroscience, Vrije Universiteit Amsterdam, Amsterdam UMC, Amsterdam Netherlands; ^9^ Department of Radiology and Nuclear Medicine, Vrije Universiteit Amsterdam, Amsterdam University Medical Center, location VUmc, Amsterdam Netherlands; ^10^ Alzheimer Center Amsterdam, Amsterdam UMC, Amsterdam Netherlands; ^11^ Erasmus University Medical Center, Rotterdam, Zuid‐Holland Netherlands; ^12^ Amsterdam Neuroscience, Vrije Universiteit Amsterdam, Amsterdam UMC, Amsterdam Netherlands; ^13^ Department of Epidemiology and Data Science, Vrije Universiteit Amsterdam, Amsterdam UMC, Amsterdam Netherlands

## Abstract

**Background:**

Hippocampal volume is an acknowledged biomarker of neurodegenerative disease, including Alzheimer’s disease (AD). However, the relationship between other subcortical brain structures and dementia risk is uncertain and may differ by disease stage. We aimed to assess the prognostic value of subcortical volumes for dementia risk across different disease stages by investigating memory clinic‐based populations and community‐dwelling individuals.

**Method:**

We included 9613 dementia‐free participants from a population‐based cohort (Rotterdam Study [n=5442, MCI:4.2%]) and two memory clinic‐based cohorts (Amsterdam Dementia Cohort [n=1964, MCI:36.1%] and the Alzheimer's Disease Research Centers of NACC: National Alzheimer’s Coordinating Center, funded by NIA/NIH Grant U24 AG072122 [n=2207, MCI:32.7%]). Mean age ranged from 62 to 65 years, and 40 to 61% were women. We performed automated segmentations of the nucleus accumbens, amygdala, caudate, hippocampus, pallidum, putamen and thalamus on T1‐MRI using Freesurfer. We determined the association between volumes of each subcortical structure and 5‐year risk of dementia in each cohort, using Cox proportional hazard models adjusted for various confounders and the other subcortical volumes.

**Result:**

During 5 years of follow‐up, dementia was diagnosed in 371 individuals in NACC, 249 in ADC, and 321 in the Rotterdam Study. Consistently across all cohorts (Figure 1), smaller hippocampal volume was significantly associated with an increased dementia risk, driven by the progression to AD dementia rather than non‐AD dementia. Smaller amygdala volume was associated with an increased dementia risk in both memory clinic cohorts, with similar effect estimates in the population‐based cohort. Smaller thalamic volume was associated with dementia risk only in the population‐based cohort, driven by the progression from normal cognition to dementia (HR: 1.73 [95%CI:1.14‐2.63]), rather than from mild cognitive impairment to dementia (HR 0.83 [0.45‐1.54]). The association between the amygdala and dementia risk was similar for AD and non‐AD dementia in both memory clinic‐based populations, while in the population‐based cohort the association was only evident in progression to AD dementia.

**Conclusion:**

Various subcortical structures play a role in the conversion to dementia. While the hippocampus and amygdala show consistent associations across populations, variation between populations for the thalamus and accumbens suggest differences in their role with underlying pathophysiology and disease stage.